# Sodium Stearate-Assisted Optimization of a Cannabidiol Dry Powder Inhaler for Enhanced Dissolution and Aerosol Performance

**DOI:** 10.3390/pharmaceutics18040512

**Published:** 2026-04-21

**Authors:** Jin-Hyuk Jeong, Jae Seok Jeong, Hyeon-Seo Moon, Jae Woon Son, Kyung Hyun Min, Dong-Wook Kim, Chang-Soo Han, Wonwoong Lee, Chun-Woong Park, Ji-Hyun Kang

**Affiliations:** 1 College of Pharmacy, Chungbuk National University, Cheongju 28160, Republic of Korea; jinddong9293@chungbuk.ac.kr; 2 Korea Zoonosis Research Institute, Jeonbuk National University, Iksan 54531, Republic of Korea; jeongjs@jbnu.ac.kr; 3 Department of Internal Medicine, Research Center for Pulmonary Disorders, Jeonbuk National University Hospital, Jeonju 54907, Republic of Korea; 4 Respiratory Drug Development Research Institute, Jeonbuk National University, Jeonju 54928, Republic of Korea; 5 School of Pharmacy, Jeonbuk National University, Jeonju 54928, Republic of Korea; mhsuou@naver.com (H.-S.M.); sonjw96@jbnu.ac.kr (J.W.S.); 6 Institute of New Drug Development, Jeonbuk National University, Jeonju 54928, Republic of Korea; 7 College of Pharmacy, Wonkwang University, Iksan 54538, Republic of Korea; pharmengin1@wku.ac.kr; 8 Department of Pharmaceutical Engineering, Cheongju University, Cheongju 28503, Republic of Korea; changsoo@cju.ac.kr; 9 College of Pharmacy, Woosuk University, Wanju 55338, Republic of Korea; wwlee@woosuk.ac.kr

**Keywords:** aerosol performance, micellization, amorphous dispersion, solubility enhancement, oxidative stability

## Abstract

**Background/Objectives**: Cannabidiol (CBD) has emerged as a potential therapeutic agent for respiratory disorders, including asthma and chronic obstructive pulmonary disease. However, its clinical translation via pulmonary delivery is limited by poor aqueous solubility, chemical instability, and low local bioavailability. This study aimed to develop and optimize a sodium stearate (NaSt)-based spray-dried dry powder inhaler (DPI) formulation to enhance the aerosol performance, dissolution, and storage stability of CBD. **Methods**: CBD microparticles were prepared by spray drying using NaSt as the primary excipient. The feed preparation method, spray-drying parameters, and CBD:NaSt ratios were systematically optimized. The resulting powders were evaluated for aerodynamic properties using cascade impaction, dissolution behavior in simulated lung fluid, solid-state characteristics, and accelerated stability under stress conditions. **Results**: The optimized formulation, SD-4, a spray-dried CBD:NaSt formulation prepared at a 20:80 weight ratio using Process B, demonstrated excellent aerosolization performance, with a fine particle fraction (FPF) exceeding 50% and a mass median aerodynamic diameter (MMAD) of 5.08 ± 0.1 μm. Dissolution testing revealed more than a three-fold increase in drug release compared with raw CBD, attributed to amorphous dispersion within the NaSt matrix and surfactant-induced micellization. Accelerated stability studies confirmed improved retention of the amorphous state and drug content, while antioxidant incorporation further reduced oxidative degradation. **Conclusions**: The NaSt-based spray-dried formulation significantly improved aerosol deposition efficiency, dissolution rate, and physicochemical stability of CBD. This formulation strategy may provide a promising platform for pulmonary delivery of poorly water-soluble compounds.

## 1. Introduction

Cannabidiol (CBD), a hydrophobic and non-psychoactive constituent of Cannabis sativa, has attracted considerable interest because of its broad pharmacological potential, including anti-inflammatory and antioxidant activities [1,2]. Recent reviews have highlighted the therapeutic potential of CBD across a wide range of disorders and delivery contexts [1,2,3]. Recent reviews have further expanded the therapeutic scope of CBD, highlighting its potential in pain management, anxiety-related disorders, and immune modulation [4,5,6]. Collectively, these findings support the broad pharmacological relevance of CBD and strengthen its potential as a multifunctional therapeutic agent.

Recent studies have demonstrated that CBD exerts anti-inflammatory, antioxidant, and anti-fibrotic effects in multiple pulmonary disease models [7,8]. It has shown therapeutic efficacy against acute lung injury [8,9,10], pulmonary fibrosis [7,11,12], and asthma [13]. Mechanistically, CBD modulates pulmonary inflammation by suppressing pro-inflammatory cytokines and oxidative stress, leading to reduced leukocyte infiltration and fibrotic remodeling [7,13]. Taken together, these findings support the potential of CBD as a therapeutic option for respiratory diseases, including asthma, chronic obstructive pulmonary disease (COPD), and pulmonary fibrosis [7,13,14].

However, despite its therapeutic potential, the clinical translation of CBD remains limited by poor aqueous solubility, low and variable bioavailability, and chemical instability [15,16,17,18,19]. Following oral administration, CBD undergoes extensive first-pass hepatic metabolism and degradation in acidic gastric environments, resulting in an oral bioavailability of only 6–19%. Consequently, it is challenging to maintain consistent plasma concentrations. Pulmonary administration has been proposed as an alternative route to circumvent these limitations, bypassing gastrointestinal degradation and first-pass metabolism while enabling rapid absorption and improved systemic exposure [3,20].

Traditional inhalation approaches, such as smoking and vaporization, have demonstrated that cannabinoids can be efficiently absorbed through the lungs. However, these routes are limited by dose variability, dependence on individual inhalation patterns, and the possible generation of harmful by-products during high-temperature processing [21,22]. These limitations highlight the need for a safer, more reproducible, and pharmaceutically stable inhalable formulation of CBD.

Dry powder inhalers (DPIs) are a promising platform for pulmonary delivery of poorly water-soluble compounds such as CBD. Because DPIs deliver solid-state formulations directly to the lungs without propellants or large solvent volumes, they offer advantages in formulation stability, dose precision, portability, and patient compliance [23,24]. They can also be engineered to achieve efficient lung deposition for both local and systemic delivery [23,24,25,26,27]. In line with this potential, recent studies have demonstrated growing interest in inhalable CBD dry powders. Tai et al. developed spray freeze-dried CBD powders containing dipalmitoylphosphatidylcholine and demonstrated improved inhalation suitability together with enhanced solubility. Komal et al. reported inhalable CBD dry powders intended for chronic obstructive pulmonary disease treatment and evaluated their in vitro aerosol and formulation characteristics. Gomes et al. also developed a spray-dried inhalable CBD powder using hydroxypropyl-β-cyclodextrin and demonstrated the feasibility of producing CBD particles with favorable aerodynamic properties and modified morphology [14,28,29]. Collectively, these studies support the feasibility of pulmonary CBD delivery, but they have mainly focused on inhalation feasibility, carrier selection, or solubility-oriented formulation strategies.

Several particle engineering approaches have been used to prepare DPI formulations, including jet milling, carrier-based blending, freeze drying or spray freeze drying, supercritical fluid-based processing, and spray drying [30,31,32]. Although each approach has specific advantages, each also presents practical limitations. Jet milling is effective for particle size reduction but often provides limited control over particle morphology and surface properties and may increase interparticle cohesion because of irregular particle surfaces. Carrier-based blending is practical and widely used, but its performance can be strongly influenced by drug-carrier adhesion and blending uniformity. Freeze drying and spray freeze drying can be useful for thermally sensitive materials, although they are generally more complex and may be less suitable for routine large-scale powder production. Supercritical fluid-based processing can generate fine particles with controlled properties, but it usually requires specialized equipment and more complicated operating conditions. In contrast, spray drying offers several formulation and manufacturing advantages, including simultaneous control of particle size, morphology, surface composition, and residual moisture content, all of which are critical determinants of aerosolization performance and storage stability in DPI systems [30,31,32]. In addition, spray drying enables one-step particle formation and facilitates the incorporation of functional excipients, making it particularly suitable for the development of inhalable formulations of poorly water-soluble drugs such as CBD.

Excipient selection is another key determinant of DPI performance. Depending on the intended formulation function, inhalation powders may contain lactose as a carrier in carrier-based systems, mannitol and trehalose as bulking or stabilizing agents, L-leucine as a dispersibility enhancer, and phospholipids or other amphiphilic materials to improve surface properties and dissolution behavior. In the present study, mannitol and L-leucine were selected as representative comparator excipients because they are widely used to improve powder handling, particle formation, and aerosol dispersion in DPI systems. In contrast, sodium stearate (NaSt), a fatty acid salt with amphiphilic and surface-active characteristics, has attracted attention as a multifunctional excipient for pulmonary drug delivery. During spray drying, NaSt preferentially migrates toward the droplet-air interface and forms a hydrophobic surface layer that can enhance powder flowability, reduce moisture-induced aggregation, and facilitate deagglomeration during aerosolization, thereby promoting efficient deposition of respirable particles [33,34,35,36]. After deposition in the pulmonary environment, NaSt can also behave as a mild anionic surfactant capable of forming micellar or vesicle-like assemblies upon contact with lung fluids [37,38]. These self-assembled structures may encapsulate hydrophobic molecules such as CBD, thereby improving wettability, dissolution, and apparent solubility while also helping to maintain CBD in an amorphous, bioavailable state [39]. Thus, unlike previously reported inhalable CBD powder systems that mainly emphasized inhalation feasibility or solubility enhancement, the present study investigated a NaSt-based spray-dried platform in which a single functional excipient could simultaneously contribute to aerosolization improvement, dissolution enhancement, and physicochemical stabilization.

Therefore, this study aimed to develop a sodium stearate-based spray-dried CBD DPI and evaluate its aerosolization performance, dissolution behavior, and stability. By systematically linking feed preparation strategy, NaSt-assisted matrix formation, hydration-induced nanostructure generation, and oxidative stability improvement, this study sought to establish a robust and scalable formulation strategy for the pulmonary delivery of highly hydrophobic cannabinoids such as CBD.

## 2. Materials and Methods

### 2.1. Materials

CBD was purchased from Cayman Chemicals (Ann Arbor, MI, USA). NaSt, mannitol, and L-leucine were purchased from Dejung (Seoul, Republic of Korea). Ultrapure water was prepared using a laboratory purification system. High-performance liquid chromatography (HPLC)-grade solvents were used for analysis, with HPLC-grade acetonitrile purchased from Honeywell Burdick & Jackson (Muskegon, MI, USA). Unless otherwise specified, all other chemicals and solvents were of analytical grade and were purchased from Merck KGaA (Darmstadt, Germany) and used without further purification.

### 2.2. Preparation of CBD Microparticles

**Figure 1:**
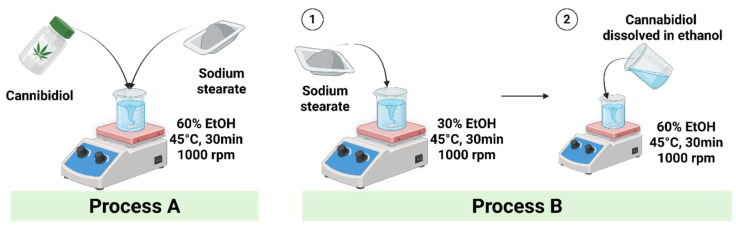
Schematic illustration of feed preparation methods prior to spray drying. In Process A, cannabidiol (CBD) and sodium stearate (NaSt) were directly mixed in 60% (*v*/*v*) ethanol under heating, followed by spray drying at 80 °C inlet temperature, 0.30 m^3^/min blower rate, and 60 kPa atomizing pressure. In Process B, ① NaSt was first dissolved in an ethanol–deionized water mixture under heating, and ② CBD predissolved in ethanol was gradually added to minimize thermal exposure, followed by spray drying at 100 °C inlet temperature, 0.45 m^3^/min blower rate, and 100 kPa atomizing pressure. Created with BioRender.com, Jeong, J. (2026) https://BioRender.com/owkkcbl.

CBD microparticles were prepared by spray drying through a sequential process consisting of feed preparation followed by spray-drying. Two feed preparation approaches differing in excipient dissolution order were evaluated to improve CBD dispersion homogeneity and formulation reproducibility. In the first approach, CBD and the selected excipient (mannitol, L-leucine, or sodium stearate) were simultaneously dissolved or dispersed in 60% (*v*/*v*) ethanol and magnetically stirred at 45 °C until a macroscopically uniform feed was obtained without visible phase separation or sedimentation. Depending on excipient type and composition, the feed appeared either as a clear solution or as a visually uniform suspension. In the second approach, sodium stearate was first dissolved in an ethanol-deionized water mixture at 45 °C, and CBD, predissolved separately in ethanol, was then gradually added under continuous stirring. After complete mixing, the ethanol content was adjusted to a final concentration of 60% (*v*/*v*). This stepwise mixing order was used to reduce CBD thermal exposure and improve dispersion uniformity. The overall feed preparation procedure is illustrated in Figure 1. The solid-state identity of any undissolved phase in the suspension was not separately characterized prior to spray drying.

For all formulations, the total solid concentration of the feed solution was fixed at 2% (*w*/*v*) prior to spray drying. Spray drying was performed using a laboratory-scale spray dryer (SD-1000; EYELA, Tokyo Rikakikai Co., Ltd., Tokyo, Japan). Two spray-drying processes were defined according to the feed preparation approach and operating parameters. In the present study, Process A and Process B were designed as two integrated manufacturing conditions, each combining feed preparation sequence and spray-drying parameters, rather than as a strict single-factor comparison. This approach was selected because feed preparation order and drying conditions were expected to interact in determining CBD dispersion state, NaSt distribution, drying behavior, and final powder properties. Accordingly, the purpose of this comparison was to identify practically distinct preparation conditions suitable for formulation screening and process selection, rather than to isolate the independent contribution of each variable. Process A, applied to formulations SD-1 to SD-3, employed the first feed preparation approach and was conducted at an inlet temperature of 80 °C, a blower rate of 0.30 m^3^/min, and an atomizing pressure of 60 kPa. Process B, applied to formulations SD-4 to SD-7, employed the second feed preparation approach and was performed at an inlet temperature of 100 °C, a blower rate of 0.45 m^3^/min, and an atomizing pressure of 100 kPa. The compositions of CBD microparticles prepared with different excipients and process conditions are summarized in Table 1. Based on Process B, additional formulations with varying CBD-to-sodium stearate ratios were prepared as listed in Table 2. All spray-dried powders were collected using a cyclone separator and stored in a desiccator until further characterization.

### 2.3. HPLC Analysis

The CBD content of each formulation was determined using a validated HPLC system (1200 Infinity; Agilent Technologies, Santa Clara, CA, USA) equipped with a quaternary pump, autosampler, column compartment, and diode array detector set to 220 nm. Separation was achieved on a C18 column (250 × 4.60 mm, 5 μm; Waters, Milford, MA, USA). An isocratic mobile phase composed of acetonitrile and deionized water (70:30, *v*/*v*) was delivered at 1.5 mL/min. The column temperature was maintained at 25 °C, and the injection volume was 10 μL. CBD quantification was carried out using an external standard approach. Standard solutions of known concentration were analyzed to construct a calibration curve from peak area versus concentration, and the resulting regression equation was used to calculate CBD levels in each formulation or analytical sample. CBD content (%) was calculated by comparing the measured CBD amount with the theoretical amount initially introduced into the formulation, as shown below. All measurements were performed within the validated linear range of the assay.

$$CBD\;content\left(\%\right)=\frac{Measured\;CBD\;amount\;in\;formulation\;or\;analytical\;sample}{Theoretical\;or\;initial\;CBD\;inpit\;amount}\times 100$$

### 2.4. Aerodynamic Performance

Aerodynamic behavior of the CBD microparticles was assessed using an eight-stage nonviable Andersen Cascade Impactor (ACI; TE-20-800; Tisch Environmental, Cleves, OH, USA) coupled to a Plastiape RS01 dry powder inhaler (Plastiape S.p.A., Osnago, Italy). The RS01^®^ device used in this work corresponded to the medium-resistance version supplied with the commercial product Fluterol^®^, with a measured pressure drop of 0.016 kPa^0.5^/(L·min^−1^) at 60 L/min using a size #3 capsule. Approximately 10 mg of each formulation was filled into hydroxypropyl methylcellulose (HPMC) capsules and dispersed through the RS01 device connected to the ACI induction port. The impactor was operated at 28.3 L/min as the predefined condition for comparative aerosol evaluation under standard ACI cutoff settings. Following dispersion, the capsule, inhaler, induction port, and each impactor stage were collected individually and washed with acetonitrile/deionized water (70:30, *v*/*v*) to recover deposited CBD. The recovered fractions were quantified by HPLC. At 28.3 L/min, the aerodynamic cutoff diameters of the ACI stages were 8.0, 6.5, 5.2, 3.5, 2.6, 1.7, 1.0, and 0.43 μm. Emitted dose (ED), fine particle dose (FPD), and fine particle fraction (FPF) were obtained from the measured CBD deposition profile. ED was defined as the percentage of the initial CBD mass loaded into the capsule that exited the capsule during actuation. FPD was defined as the total CBD mass recovered from stages 3–7, corresponding to particles in the respirable aerodynamic size range of 1.0–5.2 μm. FPF was expressed as the proportion of FPD relative to the recovered particle mass across the impactor stages. MMAD and GSD were determined according to USP General Chapter 601 [40]. MMAD was obtained from the D50% point on the logarithmic probability plot of cumulative mass fraction smaller than aerodynamic diameter, and GSD was calculated using the equation shown below.

$$ED\left(\%\right)=\;\frac{Initial\;CBD\;mass\;loaded\;into\;the\;capsule-CBD\;mass\;remaining\;in\;the\;capsule}{Initial\;CBD\;mass\;loaded\;into\;the\;capsule}\times 100$$

FPD=∑CBD mass deposited on ACI stages 3−7

$$FPF\left(\%\right)=\frac{FPD}{Mass\;of\;Particles\;in\;All\;Stages}\times 100$$

$$GSD=\sqrt{\left(D84.13\%/D15.87\%\right)}$$

### 2.5. In Vitro Dissolution Study via Franz Diffusion Cell

In vitro dissolution was evaluated using a vertical Franz diffusion cell system (Lab FINE, Gunpo, Republic of Korea). Donor and receptor chambers were separated by a Cytiva nylon membrane filter with a pore size of 0.2 μm, giving an effective diffusion area of 1.77 cm^2^. The membrane served to maintain a defined donor–receptor boundary while preventing direct transfer of undissolved particles into the receptor chamber, thereby allowing reproducible measurement of dissolved CBD over time [41,42]. Nylon was chosen because it is mechanically stable, compatible with the aqueous receptor phase, and suitable for retaining particulate matter while allowing dissolved drug to pass through [42]. The donor and receptor chamber volumes were 1 mL and 12 mL, respectively. Each formulation (10 mg) was placed in the donor chamber. The receptor medium consisted of 0.01 M phosphate-buffered saline (PBS, pH 7.4) containing 2% (*w*/*v*) Tween 80, used here as a simplified sink medium to maintain CBD solubility during comparative dissolution testing rather than as a fully biorelevant simulated lung fluid [43]. The receptor phase was maintained at 37 °C with continuous stirring. At 30, 60, 120, and 180 min, 0.5 mL aliquots were withdrawn from the receptor chamber and immediately replaced with an equal volume of fresh medium. Samples were analyzed by HPLC, and all measurements were conducted in triplicate (*n* = 3).

### 2.6. X-Ray Diffraction (XRD)

The crystalline characteristics of the drug and formulation samples were examined by X-ray diffraction (XRD) using a high-resolution diffractometer (D8 ADVANCE, Bruker, Germany). Data were collected over a 2θ range of 0–90°. The resulting diffraction patterns were used to identify characteristic crystalline reflections and to assess the physical state of the drug within the formulation.

### 2.7. Differential Scanning Calorimetry (DSC)

Thermal properties of the samples were investigated by differential scanning calorimetry (DSC) using a Discovery DSC 2500 instrument (TA Instruments, 159 Lukens Drive, New Castle, DE 19720, USA). Samples were heated from 0 to 150 °C at a rate of 10 °C/min. The thermograms were examined for melting events and other thermal transitions associated with the drug and excipients.

### 2.8. Scanning Electron Microscope (SEM)

Particle morphology was examined using scanning electron microscopy (SEM; ZEISS-GEMINI LEO 1530, Carl Zeiss AG, Oberkochen, Germany). Samples were mounted on carbon tape and coated with platinum using a Hummer VI sputter coater to a thickness of approximately 200 Å. Imaging was performed at an accelerating voltage of 3 kV with magnifications of 5000× and 20,000×.

### 2.9. Raman Imaging

Raman imaging was carried out using a random-scanning confocal Raman microscope (Ramanwalk, Nanophoton Corporation, Osaka, Japan) equipped with a 532 nm diode laser. Individual component spectra were first acquired as references, followed by Raman mapping of the powder samples. Powders were gently spread on glass microscope slides to obtain nominally flat sample surfaces. Spectra were collected over a 150 × 100 μm area with a step interval of 1.5 μm. After approximately 13 h of data acquisition, chemical distribution maps were reconstructed using the direct classical least-squares method. In the resulting images, red corresponds to CBD and green corresponds to sodium stearate.

### 2.10. Transmission Electron Microscope (TEM)

Nanostructures formed after dissolution of the DPI formulation were visualized by transmission electron microscopy (TEM) using a Hitachi H-7650 microscope equipped with a LaB_6_ electron source and operated at 80–120 kV. Samples were collected from the receptor phase of the Franz diffusion cells after 24 h release in 0.01 M PBS (pH 7.4) containing 2% (*w*/*v*) Tween 80 and were analyzed without dilution. A 5 μL aliquot was placed onto a 300-mesh carbon/Formvar-coated copper grid and allowed to adsorb for 1 min. The grid was then negatively stained with 1% (*w*/*v*) phosphotungstic acid (PTA, pH 7.0) for 30 s. Excess stain was removed using filter paper, and the grid was air-dried prior to imaging. Images were recorded using a Gatan RIO9 CMOS camera.

### 2.11. Cell Viability Study

Cytocompatibility of the sodium stearate-based CBD formulation was evaluated in alveolar epithelial cells by the 3-(4,5-dimethyl-2-thiazolyl)-2,5-diphenyl-2H-tetrazolium bromide (MTT) assay. A549 cells (CCL-185™; American Type Culture Collection, Manassas, VA, USA) were seeded in 96-well plates at 2 × 10^4^ cells per well and cultured for 24 h to allow attachment and spreading in RPMI 1640 supplemented with 10% fetal bovine serum (FBS) and 1% penicillin/streptomycin. The formulations were then dispersed in fresh RPMI 1640 containing the same supplements and applied to the cells at final concentrations of 0.4, 1.6, 6.3, 25, and 50 μg/mL for 48 h. Following treatment, the medium was removed and replaced with 100 μL of MTT solution (0.5 mg/mL), and the cells were incubated for an additional 4 h at 37 °C. The supernatant was discarded, and 100 μL dimethyl sulfoxide (DMSO) was added to dissolve the formazan crystals. After shaking the plate for 10 min, absorbance was read at 540 nm using a microplate reader (SpectraMax iD3, Molecular Devices, San Jose, CA, USA).

### 2.12. Accelerated Stability Assessment

An accelerated stability study was conducted to rapidly assess the susceptibility of the spray-dried CBD formulations to thermal and oxidative stress under forced degradation conditions. The study was performed at 40 °C and 75% relative humidity (RH) for up to 2 months, which are commonly employed accelerated conditions to predict potential instability mechanisms and formulation robustness rather than to simulate the intended long-term storage conditions of CBD. Samples were stored in amber glass vials under open conditions to allow direct exposure to heat and humidity. The CBD content was analyzed by HPLC on days 1, 3, 7, 14, 21, 30, and 60 to monitor time-dependent changes in drug content. To investigate the effects of stabilizing additives on CBD stability under accelerated conditions, additional samples were prepared by physically mixing 5% (*w*/*w*) mannitol, 5% (*w*/*w*) butylated hydroxyanisole (BHA), or 5% (*w*/*w*) butylated hydroxytoluene (BHT) with the spray-dried powder after preparation. The 5% (*w*/*w*) level was defined relative to the total solid content of the formulation. Changes in CBD content over time were compared to evaluate the stabilizing effects of these additives under thermal stress conditions.

### 2.13. Statistics

All statistical analyses were performed using GraphPad Prism 8 software (version 8.4.2; GraphPad Software, San Diego, CA, USA). Experimental data are presented as mean ± standard deviation (SD) unless otherwise stated. Statistical comparisons among multiple groups were performed using one-way analysis of variance (ANOVA), followed by appropriate post hoc multiple-comparison tests when significant differences were detected. For dissolution profiles involving two factors, formulation and time, two-way ANOVA was applied, followed by appropriate post hoc multiple-comparison tests. Comparisons between two groups were performed using an unpaired two-tailed Student’s *t*-test. A *p*-value of less than 0.05 was considered statistically significant. The levels of statistical significance are indicated in the corresponding figure legends (* *p* < 0.05, ** *p* < 0.01, *** *p* < 0.001, and **** *p* < 0.0001). The number of independent replicates (n) for each experiment is specified in the figure captions.

## 3. Results

### 3.1. Process and Excipient Screening for Spray-Dried CBD Formulations

**Figure 2:**
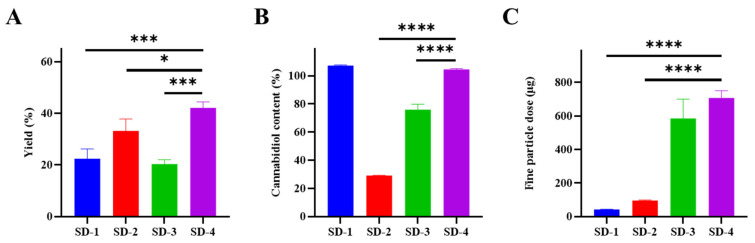
Production yield, cannabidiol (CBD) content, and fine particle dose (FPD) of spray-dried CBD formulations prepared with different excipients and process conditions (SD-1 to SD-4), showing (**A**) yield, (**B**) CBD content, and (**C**) FPD. Data are presented as mean ± SD (*n* = 3). Statistical analysis was performed using one-way ANOVA followed by Dunnett’s post hoc test with SD-4 as the control group (* *p* < 0.05, *** *p* < 0.001, **** *p* < 0.0001).

Table 1 summarizes the compositions of spray-dried CBD formulations prepared using different inhalation excipients (mannitol, leucine, and sodium stearate; NaSt) and different feed preparation strategies. To screen the influence of excipient type and process on formulation attributes, production yield, CBD content, and aerosol performance were compared across SD-1 to SD-4 (Figure 2). As shown in Figure 2A, the mannitol-based formulation (SD-1) showed a relatively low production yield of 22.40 ± 3.80%. In contrast, SD-2 (leucine-based) exhibited a higher yield of 33.20 ± 4.60%, while SD-3 (NaSt-based, Process A) resulted in a yield of 20.30 ± 1.70%. When the feed preparation and spray-drying conditions were modified (Process B), SD-4 (NaSt-based) showed an increased yield of 42.10 ± 2.30%, representing the highest yield among SD-1, SD-3, and SD-4, and exceeding that of SD-2. CBD content results are presented in Figure 2B. SD-1 exhibited the highest measured CBD content of 107.24 ± 0.53%. SD-2 showed markedly lower CBD content (29.12 ± 0.19%) compared with the other formulations. SD-3 exhibited a CBD content of 75.83 ± 3.99%, whereas SD-4 showed an increased CBD content of 104.86 ± 0.13%. CBD content values slightly exceeding 100% were observed for SD-1 and SD-4. Aerosol performance was assessed using fine particle dose (FPD), as shown in Figure 2C. SD-1 displayed the lowest FPD (42.34 ± 1.50 µg). SD-2 showed an increased FPD of 95.36 ± 3.68 µg compared with SD-1. A substantial increase in FPD was observed for the NaSt-based formulations. Specifically, SD-3 exhibited an FPD of 585.76 ± 114.9 µg, and SD-4 exhibited the highest FPD of 707.88 ± 42.38 µg among the tested formulations. For completeness, the CBD content values exceeding 100% (SD-1 and SD-4) were considered to reflect experimental and analytical variability associated with spray-dried systems, including potential weighing inaccuracy, sample heterogeneity, and variation during content analysis. Within this screening study, NaSt-based formulations (SD-3 and SD-4) consistently demonstrated higher FPD values than the mannitol- and leucine-based formulations.

### 3.2. Aerodynamic Performance and Dissolution Behavior of NaSt–CBD Formulations

**Figure 3:**
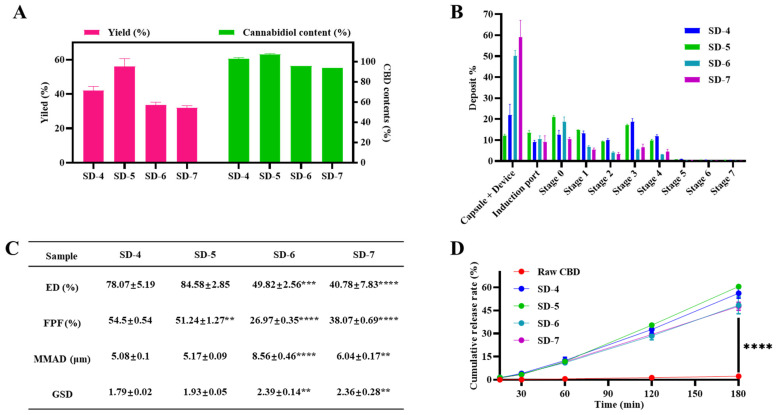
Effect of cannabidiol (CBD) to sodium stearate (NaSt) ratio on formulation performance prepared using the optimized process (Process B), showing (**A**) production yield and CBD content, (**B**) deposition profiles measured by Andersen Cascade Impactor, (**C**) aerosolization parameters, and (**D**) dissolution profiles. Data are presented as mean ± SD (*n* = 3). Statistical analysis in panel (**C**) was performed using one-way ANOVA followed by Dunnett’s post hoc test with SD-4 as the reference group (** *p* < 0.01, *** *p* < 0.001, **** *p* < 0.0001), whereas panel (**D**) was analyzed using two-way ANOVA with Tukey’s post hoc test (**** *p* < 0.0001).

Based on the optimized manufacturing strategy identified in Section 3.1 (Process B), spray-dried formulations containing different NaSt-to-CBD ratios (SD-4 to SD-7) were prepared to evaluate the composition-dependent effects on production yield, CBD content, aerosolization performance, and dissolution behavior. The compositions are summarized in Table 2, and the corresponding performance data are presented in Figure 3. As shown in Figure 3A, production yield varied depending on the NaSt content. SD-5 exhibited the highest yield (56.1 ± 4.5%), followed by SD-4 (42.1 ± 2.3%). In contrast, SD-6 (33.5 ± 1.8%) and SD-7 (32.1 ± 1.1%) showed lower yields. A decreasing trend in yield was observed with decreasing NaSt fraction. CBD content measurements showed a similar composition-dependent pattern. SD-5 and SD-4 exhibited CBD content values of 107.34 ± 0.71% and 102.96 ± 1.02%, respectively. In comparison, SD-6 and SD-7 showed lower values of 95.83 ± 0.02% and 93.64 ± 0.02%, respectively. As described in Section 3.1, CBD content values slightly exceeding 100% were attributed to experimental and analytical variability associated with spray-dried formulations.

Aerodynamic properties were evaluated using an Andersen Cascade Impactor, and the results are presented in Figure 3B,C. Fine particle fraction (FPF) and fine particle dose (FPD) differed among the formulations. SD-4 exhibited an FPF of 54.50 ± 0.54%, representing the highest or most balanced respirable fraction among the tested formulations. The corresponding FPD for SD-4 was 2228.51 ± 260.72 µg. Although SD-5 showed the highest production yield, its aerosol performance did not proportionally exceed that of SD-4. SD-6 and SD-7 exhibited reduced FPF values compared with SD-4 and showed broader aerodynamic distribution profiles. Differences in mass median aerodynamic diameter (MMAD) and geometric standard deviation (GSD) were also observed among the formulations, with SD-4 demonstrating comparatively favorable aerodynamic parameters. Overall, the aerosolization results indicated clear formulation-dependent differences in respirable dose delivery.

Dissolution profiles of raw CBD and spray-dried formulations (SD-4 to SD-7) were evaluated using 0.01 M PBS (pH 7.4) containing 2% Tween 80 as a simplified sink medium, rather than a biorelevant simulated lung fluid, to maintain CBD solubility during comparative release testing (Figure 3D) [41,43]. Raw CBD exhibited minimal dissolution over the testing period. In contrast, all spray-dried formulations demonstrated substantially enhanced dissolution behavior. At the 3 h time point, dissolution of the spray-dried formulations was up to approximately 26-fold higher than that of raw CBD. Among the spray-dried formulations, dissolution proceeded relatively rapidly during the initial phase, followed by a more gradual increase over time. Differences in dissolution extent were observed among formulations with varying NaSt content, although all spray-dried systems outperformed raw CBD under identical testing conditions.

### 3.3. Physicochemical Characterization Study

**Figure 4:**
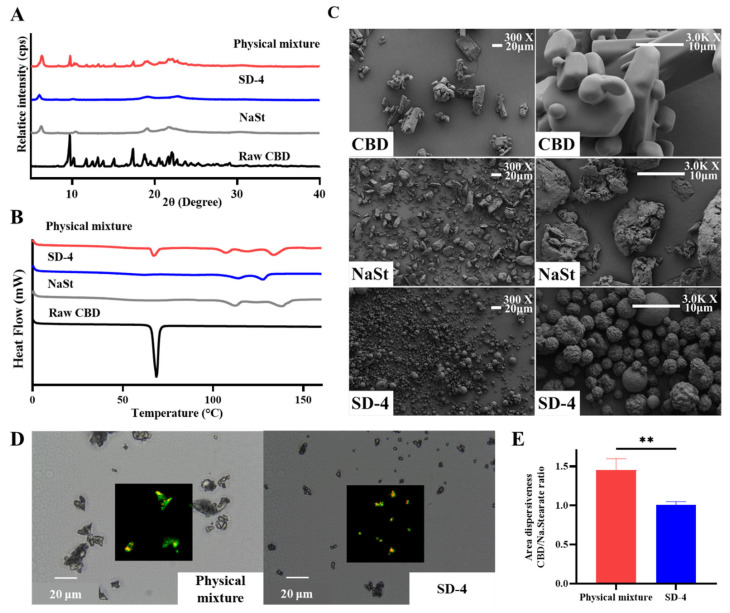
Physicochemical characterization of raw cannabidiol (CBD), sodium stearate (NaSt), physical mixture, and SD-4 formulation showing (**A**) X-ray diffraction patterns, (**B**) differential scanning calorimetry thermograms, (**C**) scanning electron microscopy images at ×300 and ×3000 magnification, (**D**) Raman mapping images, and (**E**) distribution ratios of CBD and NaSt analyzed by Student’s *t*-test (** *p* < 0.01).

The solid-state and morphological properties of the optimized spray-dried formulation (SD-4) were characterized using X-ray diffraction (XRD), differential scanning calorimetry (DSC), scanning electron microscopy (SEM), and Raman mapping analysis. For comparison, raw CBD, NaSt, and a physical mixture (CBD:NaSt = 2:8, *w*/*w*) corresponding to the same composition as SD-4 were analyzed. The results are presented in Figure 4. The XRD patterns are shown in Figure 4A. Raw CBD exhibited multiple sharp diffraction peaks characteristic of its crystalline structure. In contrast, SD-4 did not display the characteristic crystalline peaks of CBD. Instead, SD-4 showed a halo pattern, indicative of reduced long-range molecular order. NaSt exhibited its own distinct diffraction pattern. The physical mixture retained the characteristic diffraction peaks of raw CBD, indicating that simple blending did not alter the crystalline structure of CBD under the tested conditions.

DSC thermograms are presented in Figure 4B. Raw CBD exhibited a sharp endothermic melting peak corresponding to its crystalline melting temperature. This melting peak was clearly observed in the thermogram of the physical mixture as well. In contrast, SD-4 did not exhibit the characteristic melting endotherm of CBD within the scanned temperature range. NaSt showed its own thermal transition profile, which was distinguishable from that of CBD. The absence of the CBD melting peak in SD-4 was consistent with the XRD findings.

SEM images are shown in Figure 4C. Raw CBD appeared as irregularly shaped particles with heterogeneous size distribution and evident aggregation. NaSt also displayed irregular and aggregated morphology. In contrast, SD-4 consisted predominantly of spherical microparticles with relatively smooth surfaces and a comparatively more uniform particle size distribution. Reduced visible aggregation was observed in SD-4 relative to the raw materials.

Raman mapping was performed to assess the spatial distribution of CBD within the particles (Figure 4D,E). Characteristic Raman peaks of CBD and NaSt were used to construct chemical distribution maps. In the physical mixture, CBD-associated Raman signals appeared localized and heterogeneously distributed, consistent with the presence of discrete CBD particles. In contrast, SD-4 exhibited a more homogeneous distribution of CBD-associated Raman signals throughout individual microparticles. Quantitative mapping analysis indicated a CBD-to-NaSt distribution ratio of approximately 1.01 in SD-4, suggesting a uniform composition within the analyzed regions.

### 3.4. Morphological Study in Dissolution Medium

**Figure 5:**
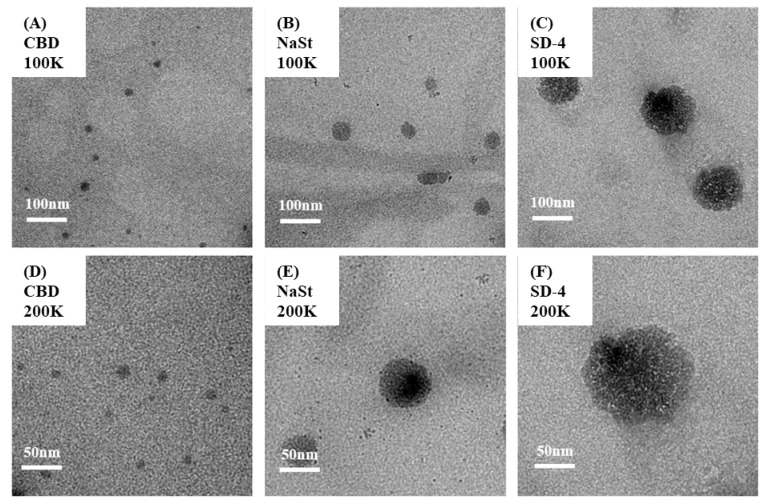
Transmission electron microscopy images of nanostructures observed after 24 h dissolution in 2% Tween 80 and 0.01 M phosphate-buffered saline (pH 7.4) showing raw cannabidiol (**A**,**D**), sodium stearate (**B**,**E**), and SD-4 formulation (**C**,**F**) captured at 100,000× magnification (**A**–**C**) and 200,000× magnification (**D**–**F**).

To investigate the structural characteristics of the samples under dissolution conditions, transmission electron microscopy (TEM) analysis was performed after dispersion in 0.01 M PBS (pH 7.4) containing 2% Tween 80, which was used as a simplified sink medium, at 37 °C. Representative TEM images are shown in Figure 5. Raw CBD, when dispersed in the dissolution medium, formed predominantly small nanoscale structures with an approximate diameter of ~10 nm. These structures appeared relatively uniform and were observed throughout the field of view. No large aggregates or vesicle-like assemblies were detected under the examined conditions. NaSt alone, dispersed under the same experimental conditions, produced spherical aggregates with sizes ranging approximately from 50 to 100 nm. These aggregates were larger than the nanoscale structures observed for raw CBD. The structures appeared as electron-dense clusters with relatively defined boundaries, and the size distribution was broader than that of raw CBD. In contrast, the spray-dried formulation SD-4 generated larger nanostructures in the dissolution medium. TEM images revealed vesicle-like assemblies with diameters exceeding 100 nm. These structures were substantially larger than those observed for either raw CBD or NaSt alone. The vesicle-like morphology was characterized by spherical outlines with lighter internal regions and darker peripheries, suggesting bilayer- or shell-like structural features. The nanostructures observed for SD-4 appeared more heterogeneous in size than the smaller assemblies seen for raw CBD. In addition, the overall structural organization in SD-4 differed distinctly from that of NaSt alone, with the presence of larger and more complex assemblies. No obvious crystalline particles were observed in the SD-4 dispersion under the examined conditions. The morphology remained predominantly nanoscale and vesicle-like rather than particulate crystalline fragments. Overall, TEM analysis revealed clear differences in nanostructural organization among raw CBD, NaSt, and SD-4 when dispersed in the dissolution medium, with SD-4 forming the largest vesicle-like assemblies among the tested samples. Because the primary objective of this section was to obtain qualitative morphological evidence of hydration-induced nanostructure formation, additional colloidal characterization, including zeta potential measurement, was not performed in the present study. Such analysis would be useful in future work to further elucidate the dispersion stability and interfacial properties of the formed structures.

### 3.5. Cytotoxicity Study in A549 Cells

**Figure 6:**
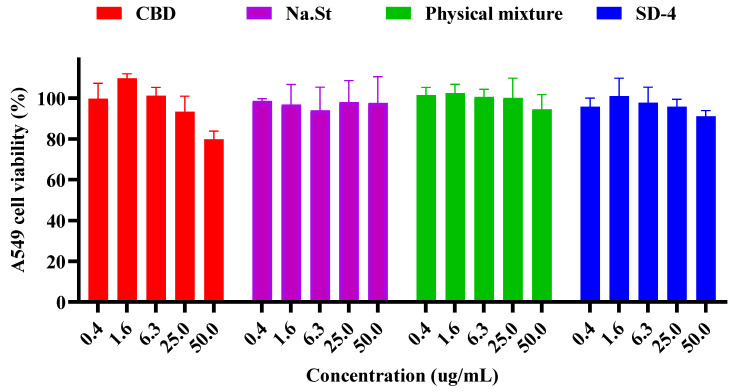
Cell viability of A549 cells after 48 h exposure to sodium stearate-based cannabidiol formulations at different concentrations determined by MTT assay (mean ± SD, *n* = 6).

The cytotoxicity of raw CBD, NaSt, the physical mixture (CBD:NaSt = 2:8, *w*/*w*), and the optimized spray-dried formulation (SD-4) was evaluated in A549 human lung epithelial cells using an MTT assay following 48 h exposure. Test concentrations ranged from 0.4 to 50 μg/mL. The results are presented in Figure 6. For raw CBD, cell viability decreased in a concentration-dependent manner. At the highest tested concentration (50 μg/mL), cell viability was approximately 79.8% relative to untreated control cells. At lower concentrations, the reduction in viability was less pronounced, and cell viability remained above 85% across most of the tested range. NaSt alone maintained cell viability comparable to that of untreated controls across the entire concentration range (0.4–50 μg/mL). No significant concentration-dependent reduction in viability was observed under the tested conditions. Similarly, the physical mixture and the spray-dried formulation SD-4 did not show a marked decrease in cell viability up to 50 μg/mL. Cell viability remained close to control levels across the tested concentration range for both formulations. It should be noted that both the physical mixture and SD-4 contain 20% (*w*/*w*) CBD. Therefore, a nominal formulation concentration of 50 μg/mL corresponds to an effective CBD concentration of approximately 10 μg/mL. Under these conditions, no significant reduction in cell viability was observed for the formulation groups. Across all tested samples except raw CBD at the highest concentration, cell viability values remained generally above 80%, indicating no severe cytotoxic response under the experimental conditions employed.

### 3.6. Accelerated Stability Study

**Figure 7:**
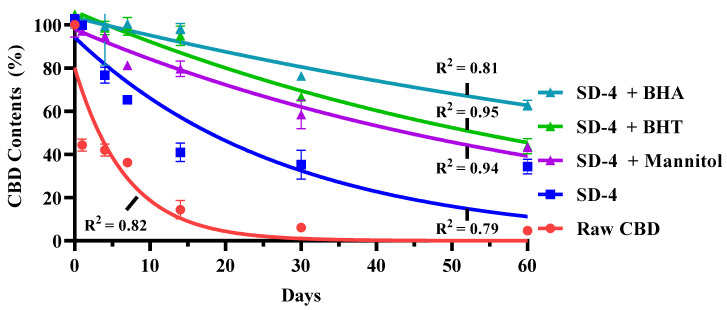
Accelerated stability profiles of cannabidiol dry powder inhaler formulations containing different stabilizing excipients stored at 40 °C and 75% relative humidity for 60 days showing measured cannabidiol content (symbols, mean ± SD, *n* = 3) and logarithmic regression fits (solid lines).

The chemical stability of raw CBD and the optimized spray-dried formulation (SD-4) was evaluated under accelerated storage conditions (40 °C/75% relative humidity) for 60 days. CBD content was quantified at predetermined time points, and the results are presented in Figure 7. Raw CBD exhibited rapid degradation under the accelerated conditions. At day 60, only approximately 4.7% of the initial CBD content remained relative to the baseline value (day 0). A steep decline in CBD content was observed within the first 14 days, followed by continued degradation throughout the storage period. In contrast, SD-4 demonstrated improved retention of CBD content compared with raw CBD. At day 60, SD-4 retained approximately 34.4% of its initial CBD content based on experimentally measured mean values. Although a reduction in CBD content was also observed in SD-4 during the early storage phase, the extent of degradation was markedly lower than that observed for raw CBD over the entire study period. For visualization of degradation trends, logarithmic regression curves (Y = a + b·ln(X)) were applied to the data points in Figure 7. These fitted curves were used to illustrate the overall degradation pattern and do not represent the exact experimentally measured values at each time point. The experimentally quantified value of 34.4% at day 60 for SD-4 corresponds to the measured mean rather than the regression estimate. In addition to SD-4, formulations supplemented with different stabilizing excipients were evaluated, including mannitol, butylated hydroxyanisole (BHA), and butylated hydroxytoluene (BHT). Mannitol-containing formulations showed only modest differences in CBD retention compared with SD-4. In contrast, formulations containing BHA or BHT exhibited higher CBD content throughout the 60-day storage period compared with SD-4 without antioxidant supplementation. Among the tested antioxidants, BHT showed the highest CBD retention under accelerated conditions. Across all formulations, a greater degree of CBD degradation was observed during the initial storage phase (up to approximately 14 days), followed by a comparatively slower rate of decline thereafter. Overall, under accelerated stress conditions, SD-4 demonstrated substantially improved CBD retention compared with raw CBD, and antioxidant incorporation further enhanced chemical stability over the 60-day storage period.

## 4. Discussion

The present study systematically investigated excipient selection, process optimization, and compositional effects for the development of a spray-dried CBD dry powder inhaler (DPI). The results collectively demonstrate that NaSt-assisted matrix engineering enables simultaneous improvement in aerosolization performance, dissolution behavior, and chemical stability relative to raw CBD and alternative excipient systems.

Among the screened excipients, NaSt-containing formulations consistently exhibited substantially higher FPD values than mannitol- and leucine-based systems. While mannitol is widely used in inhalation formulations due to its crystallinity and dispersibility, its hygroscopic nature and limited surface-modifying capability may promote interparticle cohesion under spray-drying conditions. In the present study, the mannitol-based formulation (SD-1) showed low FPD despite acceptable CBD content, indicating that drug loading alone does not guarantee respirable dose delivery. Leucine is known to migrate toward the droplet surface during spray drying and can improve powder dispersibility. However, in this study, the leucine-based formulation (SD-2) exhibited markedly low CBD incorporation efficiency. This suggests that the physicochemical compatibility between CBD and the selected excipient strongly influences drug recovery and aerosol performance. In contrast, NaSt-based formulations (SD-3 and SD-4) showed dramatic increases in FPD, suggesting that NaSt effectively reduces interparticle cohesive forces. This behavior is consistent with the known function of stearate excipients as surface modifiers in DPI systems. The improvement in FPD without proportional increases in particle mass output indicates that NaSt influences aerosol dispersion efficiency rather than merely increasing powder recovery.

Modification of feed preparation and spray-drying parameters (Process B) resulted in simultaneous improvements in yield, CBD content, and FPD. Reducing thermal exposure during feed preparation and adjusting inlet temperature and airflow likely influenced droplet drying kinetics and surface enrichment behavior. Although detailed interfacial composition analysis was not performed, the improved performance suggests that process conditions modulate excipient migration and drug incorporation within spray-dried particles. These findings highlight the importance of coupling excipient selection with optimized manufacturing parameters. In spray-dried inhalation systems, formulation performance is governed not only by composition but also by droplet drying dynamics and surface-active component redistribution.

All spray-dried formulations exhibited markedly enhanced dissolution compared with raw CBD, even under sink conditions maintained by 2% Tween 80. In the present study, Tween 80 was included primarily as a solubilizing aid to maintain analytical sink conditions for CBD, given its extremely low aqueous solubility. Accordingly, the dissolution experiment was designed to compare the relative release behavior of raw CBD and spray-dried formulations under conditions that allowed reliable quantification of dissolved drug over time. The magnitude of improvement (up to approximately 26-fold at 3 h) suggests that particle size reduction alone cannot fully account for the observed effect. Solid-state analyses confirmed amorphization of CBD in SD-4, as evidenced by disappearance of crystalline diffraction peaks (XRD) and melting endotherms (DSC). Amorphous dispersion increases apparent solubility and reduces lattice energy barriers to dissolution. Raman mapping further demonstrated homogeneous molecular distribution of CBD within the NaSt matrix, indicating effective drug–excipient integration during spray drying. Uniform dispersion likely reduces localized supersaturation and recrystallization tendencies upon hydration. TEM observations revealed formation of vesicle-like nanostructures in the dissolution medium for SD-4, which were substantially larger and structurally distinct from assemblies formed by NaSt alone. Although the precise internal organization was not resolved, these structures suggest co-assembly between CBD and NaSt upon hydration. Given the extreme hydrophobicity of CBD (LogP 6–7), incorporation into NaSt-associated assemblies may promote wettability and maintain CBD in a dispersed state, thereby contributing to sustained dissolution enhancement. Collectively, dissolution improvement appears to arise from a combination of (i) amorphization, (ii) homogeneous drug dispersion, and (iii) hydration-induced nanostructure formation.

CBD is known to be chemically unstable under oxidative and thermal stress. Under accelerated conditions (40 °C/75% RH), raw CBD underwent rapid degradation, retaining less than 5% of its initial content at day 60. SD-4 demonstrated a more than seven-fold improvement in CBD retention compared with raw CBD. The two-phase degradation profile—rapid initial decline followed by slower degradation—suggests that surface-exposed CBD may be more susceptible during early storage, while the remaining fraction is relatively protected within the matrix. Although direct surface composition measurements were not conducted, NaSt likely accumulates at the droplet–air interface during spray drying due to its surface-active properties. Such surface enrichment could form a partially protective layer that reduces moisture penetration and limits molecular mobility. However, substantial degradation still occurred under severe stress conditions, indicating that NaSt alone does not fully prevent oxidative pathways. Incorporation of phenolic antioxidants (BHA and BHT) significantly improved CBD retention, supporting the hypothesis that radical-mediated oxidation is a dominant degradation mechanism. The enhanced protection observed with BHT further suggests differences in antioxidant efficiency under the tested conditions. It should be emphasized that the antioxidant concentrations used in this study were proof-of-concept levels rather than clinically optimized doses. Therefore, further evaluation using pharmaceutically relevant concentrations and inhalation-compatible stabilizers is required.

The NaSt-based spray-dried formulation did not induce significant cytotoxicity in A549 cells across the tested concentration range. While raw CBD showed a modest concentration-dependent reduction in viability at high concentration, both the physical mixture and SD-4 maintained viability comparable to controls up to 50 μg/mL (equivalent to ~10 μg/mL CBD in the formulation groups). These findings suggest that incorporation of CBD within the NaSt matrix does not exacerbate acute cytotoxic responses under in vitro conditions. Nevertheless, in vitro monolayer models cannot fully replicate the pulmonary epithelial barrier, mucus layer, or immune interactions. Comprehensive safety evaluation, including long-term exposure and in vivo studies, remains necessary.

Pulmonary delivery offers a strategy to bypass first-pass metabolism and achieve rapid systemic exposure. However, successful translation requires optimized aerodynamic performance, dissolution behavior in lung fluid, and chemical stability. The present findings indicate that NaSt-assisted spray drying can address multiple formulation challenges simultaneously. Importantly, the optimal formulation (SD-4) achieved a balance between manufacturability, respirable dose delivery (FPF > 50%), enhanced dissolution, and improved stability relative to raw CBD. Rather than functioning solely as an anti-agglomeration agent, NaSt appears to act as a multifunctional matrix former that influences particle surface properties, solid-state characteristics, and hydration behavior. This multifunctional role may be particularly advantageous for highly hydrophobic cannabinoids and other poorly water-soluble compounds.

In the context of recent advances in inhalable CBD powders, different carrier and particle-design strategies have been explored to improve pulmonary delivery feasibility. However, those reports have primarily emphasized aerodynamic suitability, carrier selection, or solubility-oriented formulation design [14,28,29]. In contrast, the present study highlights a NaSt-based spray-dried system in which a single functional excipient simultaneously contributed to aerosolization improvement, dissolution enhancement, and physicochemical stabilization. In addition, the present work systematically connected feed preparation strategy, NaSt-assisted matrix formation, hydration-induced nanostructure generation, and oxidative stability behavior within one formulation platform. These features distinguish the current system from previously reported inhalable CBD powders and support its value as a multifunctional formulation strategy for highly hydrophobic cannabinoids.

Several limitations should be acknowledged. First, direct quantification of surface enrichment and molecular interactions was not performed. Second, dissolution testing was conducted under sink conditions containing surfactant, which may not fully represent the pulmonary microenvironment. In particular, Tween 80 was used to maintain CBD solubility and enable comparative release analysis, but no separate surfactant-free dissolution experiment was performed to determine how strongly the observed release behavior depended on the presence of surfactant. Third, the comparison between Process A and Process B was based on integrated preparation conditions in which feed preparation sequence and multiple spray-drying parameters were changed simultaneously. Therefore, the individual contribution of each processing variable to formulation performance could not be rigorously isolated. Fourth, aerodynamic assessment was conducted at a single ACI flow rate of 28.3 L/min, and thus flow-rate-dependent aerosolization behavior relevant to different inhalation efforts and device resistance was not evaluated. Fifth, additional colloidal characterization, including zeta potential measurement, was not performed and would be useful for further elucidating the stability and interfacial properties of the hydration-induced nanostructures. Sixth, antioxidant concentrations used for proof-of-concept stability enhancement exceed typical pharmaceutical levels and require optimization for translational relevance. Future studies should therefore include detailed surface analysis such as XPS or ToF-SIMS, comparative dissolution testing in surfactant-containing and surfactant-free or more pulmonary-relevant media, systematic process optimization using Design of Experiments (DoE) or factor-by-factor analysis, evaluation at multiple ACI flow rates, zeta potential-based colloidal stability assessment, long-term stability under ICH conditions, and in vivo pharmacokinetic evaluation.

Overall, this study demonstrates that NaSt-assisted spray drying enables integrated optimization of aerosol performance, dissolution enhancement, and partial chemical stabilization of CBD. These findings support surfactant-mediated matrix engineering as a promising formulation strategy for pulmonary delivery of highly hydrophobic therapeutic agents.

## 5. Conclusions

This study demonstrates a NaSt-based spray-dried DPI as an effective formulation strategy to overcome the poor solubility, instability, and limited pulmonary bioavailability of CBD. The optimized NaSt–CBD formulation exhibited a fine particle fraction exceeding 50%, uniform spherical morphology, and excellent dispersibility, enabling efficient pulmonary delivery.

NaSt functioned as a multifunctional excipient by simultaneously improving powder aerosolization and drug dissolution. Its surface-active properties reduced particle cohesion and moisture-induced aggregation during spray drying and storage, while facilitating the formation of solubilizing nanostructures upon contact with pulmonary fluid, thereby enhancing CBD wettability and dissolution. These dual physical and functional roles distinguish NaSt from conventional hydrophilic excipients such as mannitol and leucine.

Furthermore, the incorporation of antioxidants, particularly BHT, significantly enhanced the chemical stability of CBD under accelerated conditions without compromising aerosol performance. The NaSt–CBD formulation also showed good cytocompatibility in A549 cells, supporting its suitability for pulmonary administration.

Overall, this work highlights the rational integration of a surfactant-like excipient with a highly lipophilic drug as a robust design principle for inhalation formulations. The NaSt-assisted platform offers a versatile and scalable approach for the pulmonary delivery of poorly water-soluble and oxidation-prone therapeutics, supporting the development of stable, efficient, and clinically translatable cannabinoid-based inhalation therapies.

## Figures and Tables

**Table 1:** Formulations of cannabidiol dry powder inhalers prepared using different excipients and process conditions expressed as weight percentage (%).

Sample	SD-1	SD-2	SD-3	SD-4
Cannabidiol	20	20	20	20
Mannitol	80	-	-	-
Leucine	-	80	-	-
Sodium stearate	-	-	80	80
Process	Process A	Process A	Process A	Process B

**Table 2:** Formulations of cannabidiol dry powder inhalers with varying sodium stearate to CBD ratios expressed as weight percentage (%).

Sample	SD-4	SD-5	SD-6	SD-7
Cannabidiol	20	10	30	40
Sodium stearate	80	90	70	60
Process	Process B	Process B	Process B	Process B

## Data Availability

The raw data supporting the conclusions of this article will be made available by the authors on request.

## Abbreviations

The following abbreviations are used in this manuscript:

ACI	Andersen Cascade Impactor
BHA	Butylated hydroxyanisole
BHT	Butylated hydroxytoluene
CBD	Cannabidiol
COPD	Chronic obstructive pulmonary disease
DSC	Differential scanning calorimetry
DMSO	Dimethyl sulfoxide
DPI	Dry powder inhaler
DW	Distilled water
ED	Emitted dose
FPD	Fine particle dose
FPF	Fine particle fraction
GSD	Geometric standard deviation
HPLC	High-performance liquid chromatography
MMAD	Mass median aerodynamic diameter
MTT	3-(4,5-dimethyl-2-thiazolyl)-2,5-diphenyl-2H-tetrazolium bromide
NaSt	Sodium stearate
PBS	Phosphate-buffered saline
PTA	Phosphotungstic acid
RH	Relative humidity
SD	Standard deviation
SEM	Scanning electron microscopy
TEM	Transmission electron microscopy
UV	Ultraviolet
XRD	X-ray diffraction
